# Laryngology

**Published:** 1894-12

**Authors:** Barclay J. Baron


### LARYNGOLOGY.

During the past few months the Medical Journals all over the world have published striking cases illustrating the action of antitoxin in the treatment of diphtheria. When we remember, however, that the teaching of all recent experience goes to show that the absolute and infallible diagnosis of true diphtheria depends on a bacteriological investigation which only experts in such work are fitted to make, it behoves us to be very cautious in accepting the statements about the action of any remedy in this disease. Antitoxin has, however, been tested on a large scale and with the bacteriological check in every case, by Ehrlich, Kossel, and Wassermann,[1] who treated 220 cases and cured 76.4 per cent. Of 30 of the cases in which tracheotomy was necessary, 55.1 per cent. were cured. All the children who were inoculated on the first day of the disease were rescued, and the mortality steadily increased with every day that the disease continued untreated by the specific until on the fifth day it was 56 per cent.

Roux[2] has treated 300 cases of true diphtheria between February 1st and July 24th, 1894, with 78 deaths, *i.e.* 26 per cent., whereas the average mortality in the same hospital under

[1] *Deutsche. med. Wchnschr.*, 1894, No. 16. Abs. in *J. Laryngol.*, vol. viii., 1894, p. 592.
[2] *Lancet*, 1894, vol. ii., p. 675.

the most approved older methods had previously been 50 per cent. A large number of children who were admitted into the diphtheria ward as suffering from that disease, but in whom the specific bacillus was not found, were inoculated pending the bacteriological investigation, and none of them took the infection although freely exposed to it. The dose used was 20 cubic centimetres on admission, and in most cases 10 to 20 cubic centimetres twenty-four hours afterwards, and this usually completed the cure. The pulse and temperature were taken as the guides for the repetition of the injection, and so long as the temperature did not fall below 100.4° it was repeated. The maximum quantity used in any case was 125 cubic centimetres. The false membrane ceases to grow within twenty-four hours of the first injection, and detaches itself usually within thirty-six to forty-eight hours, at latest on the third day, and it ceases to produce any bacillus culture from the third to the fifth day. In the light cases the fall of temperature occurs very soon after inoculation; in the severe ones it only begins after the second or third dose, and resolution by lysis is the rule. The pulse remains quick for days. If the streptococcus is associated with the specific bacillus the cases are more serious and several injections may be necessary.

The cases of pure diphtheritic croup numbered 49, with 15 deaths; *i.e.*, 30.9 per cent. Deducting four cases that came in so ill that they died in less than twenty-four hours, we have 45 cases with 11 deaths; *i.e.*, 24.4 per cent. The cases of associated croup numbered 72, and deducting ten cases that were in the ward less than twenty-four hours, 62 cases with 31 deaths; *i.e.*, 50 per cent. All these 121 children were tracheotomised, and 56 died, *i.e.* 46 per cent., as against 68 per cent. under older methods of treatment. We know that the cases in which tracheotomy is necessary are much more serious than those in which it is not necessary, and it is very interesting to note that whereas at least 50 per cent. of all cases of diphtheria were tracheotomised when treated by the older methods, only 40 per cent. were tracheotomised of those treated with antitoxin.

Later statistics published in our own and foreign medical journals go to show that the mortality rate is distinctly less than that shown by Roux's cases.

There is, unfortunately, great difficulty in obtaining a supply of antitoxin; the British Institute of Preventive Medicine is quite unable to keep pace with the demand. Sir Joseph Lister's appeal for £2,000 to enable the Institute to place an adequate supply at the service of all medical men who require it, met with a tardy response, very different from the generous contributions of French municipalities in aid of Roux's treatment of his numerous cases, and of the Pasteur Institute. Another important point is that Behring[1] denies that Aronson's serum

[1] See *Deutsche. med. Wchnschr.*, 1894, Nos. 21, 15, quoted in *J. Laryngol.*, July and September, 1894.

obtained through Schering is as powerful as his own, although the latter serum has been used at St. Bartholomew's Hospital, and by some medical men in their private practice. It is, therefore, of prime importance that the supply should be made adequate to the demand, and that it should be of one standard quality and strength, which can only be accomplished by ample funds being placed at the disposal of the Institute of Preventive Medicine, or some such bureau of distribution, and if necessary the Local Government Board ought to step in and help.

A most exhaustive report on diphtheria and pseudo-diphtheria has been published on behalf of the New York City Health Department by Drs. Park and Beebe.[1] In it they state that 5,611 cases of suspected diphtheria were bacteriologically examined during the year ending May 4th, 1894, and of these the Loeffler bacillus was found in 3,255; *i.e.*, 58 per cent. It was not found in 1,540 cases, *i.e.* 27 per cent.; and it was doubtful in 816 cases, *i.e.* 15 per cent., because the exudate was either obtained too late in the disease, *e.g.* after the fourth day, or was allowed to dry, or was imperfectly taken from the throat, or the culture media were allowed to become contaminated. Of all the cases of acute laryngitis in children that have been examined in the bacteriological laboratory during the past ten months, about 80 per cent. were undoubtedly diphtheritic, and of the remaining 20 per cent. only 14 per cent. were certainly not diphtheritic. They show, conclusively, that false membrane may be quite absent from the pharynx and tonsils, and yet the case be truly diphtheritic.

It has been found that streptococci are regularly present in the throats of persons living in large cities, and that other forms of cocci, especially the pneumococci and staphylococci, are apt to be associated with them. So long as the mucous membrane is healthy, they create no disturbance; but damage to it by cold, specific fevers, &c., at once enables them to attack the mucous surface and cause an inflammation. This results commonly in an exudate appearing, on the tonsils by preference; but it may extend into the pharynx, larynx, and nostrils. The mortality is greatly less than it is in true diphtheria, and it is seldom, if ever, communicable.

I have just had under treatment a case of this kind, with "patches" on both faucial tonsils, pharynx and lingual tonsil, persisting for weeks; but the patient has not felt ill, has had no rise of temperature, and is evidently not infectious to other people.

Drs. Park and Beebe find that there are at least two kinds of bacteria to which the name "pseudo-diphtheria bacillus" has been given: 1. Bacilli which differ in no way from genuine diphtheria bacilli, except that they lack virulence. As Roux, Yersin, and Fränkel have pointed out, this loss of virulence occurs naturally at times, and can also be produced artificially.

[1] *J. Laryngol.*, vol. viii., 1894., p. 699.

2. There are the bacilli described by Escherich, which are uniform in their peculiarities in staining, size, shape, and the production of an alkali instead of an acid, to which alone the name "pseudo-diphtheria bacilli" should be given.

The Loeffler bacillus can live for long periods outside the body: *e.g.*, on blood serum, 155 days (Hofmann); on blood serum, seven months (Loeffler); in gelatine, eighteen months (Klein); in dried membrane, twenty weeks (Roux and Yersin); on a child's toy kept in a dark place, for five months. A number of cases were met with where infected bedding or clothing had spread the disease.

Since the bacteriological investigation of all cases in which diphtheria is suspected is so extremely important; in fact, since it alone enables the sanitary authority in a city to know the extent of the disease, if any be present, to which the city is subject, and since it alone enables private practitioners and the staffs of hospitals to know what case is truly diphtheritic, it is urgently necessary that the Government or municipal authorities should appoint officers and equip laboratories for the special purpose of investigating suspected cases. In fact, every medical man should be supplied with the necessary apparatus for collecting material, to be sent to the laboratory for cultivation and examination; a report to follow this as rapidly as possible.[1] This is being done in America on a large scale. I believe that a resolution of the British Laryngological Association has been submitted to the Local Government Board on the subject, and it is worth the attention of all medical associations in our country.

Barclay J. Baron.

[1] Mr. F. Wallis Stoddart, public analyst for Bristol, is prepared upon application to supply sterilised tubes (with swab) to any medical man who has a doubtful case. When these are returned to him he will submit them to bacteriological investigation, and furnish a report.

Beginning in January, Dr. D. S. Davies, the medical officer of Health for Bristol, will supply "culture outfits" and, in the Sanitary Authority's laboratory, examine cultures from suspected cases of diphtheria.—Ed.

